# Anti-microbial effect of probiotic versus silver nanoparticle irrigant

**DOI:** 10.6026/973206300211295

**Published:** 2025-05-31

**Authors:** Neelanjana Majee, Prasanti Kumari Pradhan, Rashmi Rekha Mallick, Neha Agrawal, Ashtha Arya, Deepashri Arvind Tekam

**Affiliations:** 1 Department of Conservative Dentistry and Endodontics, Kalinga institute of dental sciences KIIT deemed to be University, Bhubaneswar, India; 2 Department of Conservative Dentistry and Endodontics, SCB Dental College, Odisha University of Health Sciences, Bhubaneswar, India; 3 Department of Biochemistry, Index medical college hospital and research center, Indore, Madhya Pradesh, India; 4 Department of Conservative Dentistry and Endodontics, SGT Dental College, Hospital and Research Institute, SGT University, Gurugram, Haryana, India; 5 Department of Conservative Dentistry and Endodontics, SharadPawar Dental College and Hospital, Sawangi, Wardha, Maharashtra, India

**Keywords:** Nanoparticles, probiotic irigant, antimicrobial efficacy, *E. faecalis*, root canal disinfection

## Abstract

Disinfection of root canal system is a big challenge in the path of successful long-term treatment outcomes. 32 nonpathogenic extracted human teeth with single roots were collected for the study and were inoculated with Enterococus faecalis (*E. faecalis*) and later treated with silver nitrate solution and probiotic solution. The mean values of the *E. faecalis* count of probiotic irrigant (mean = 1.12) is lesser than that of silver nanoparticle irrigant (mean = 2.39). The probiotic irrigant had better antimicrobial efficacy than silver nanoparticle irrigant against *E. faecalis*.

## Background:

The primary objective in treating periapical disease is the thorough elimination of complex microbial infections from the root canal system, which is essential for successful healing and long-term treatment outcomes [1]. The root canal disinfection protocol is sometimes strenuous due to the anatomical variations and the adherent biofilm structure of microorganisms [2]. *E. faecalis* is the most resistant endodontic pathogen, often causing secondary infections. Root canal disinfection is achieved mechanically or chemically using antimicrobial solutions [3]. The irrigating solutions pumped in to the root canals with the help of syringe help in eliminating the bacteria and dissolve organic and inorganic tissue [4]. Sodium hypochlorite (NaOCl) is the only irrigant that dissolves the entire organic content of the root canal, eliminating biofilm and necrotic tissue. However, if pushed beyond the apex, it can cause severe pain and swelling due to its cytotoxic effects on surrounding periapical tissue [5]. This being the biggest demerit of sodium hypochlorite leads to the search of an alternative biocompatible irrigating solution which should balance between safety and antimicrobial effectiveness [6, 7]. Nanoparticles are innovative antimicrobial delivery systems that improve antibacterial agents in root canal therapy. Their small size (100 nm), high surface area-to-mass ratio, increased reactivity and greater charge density enhance their interaction with pathogens, boosting antibacterial action [8]. Numerous investigations have demonstrated the connection between silver nanopariticle (AgNP) antimicrobial properties and oxidative dissolution and silver ion release. In dentistry, probiotics have recently been employed for infection prevention or treatment. According to World Health Organization, "probiotics" are microorganisms which when given in required amounts benefits the host [9]. Kumar *et al.* reported that probiotics exhibit antimicrobial effects primarily through the production of substances such as hydrogen peroxide, lactic acid and bacteriocins, which contribute to their ability to inhibit the growth of harmful microorganisms [10]. Therefore, it is of interest to compare the effect of probiotic irrigant with that of silver nanoparticle irrigant against *E. faecalis*.

## Methodology:

## Preparation of the teeth sample:

32 nonpathogenic extracted human teeth with single roots were collected for the study. They were kept in 3 % sodium hypochlorite (PARCAN, Septodont Healthcare India Pvt. Ltd) to remove the organic debris and the calculus was removed by scaling. After that they were then soaked in the saline until instrumentation of the teeth. Access cavities were prepared and working lengths determined. Biomechanical preparation was done using rotary files (Endostar E3 Azure®, POLDENT), with root canals shaped to 25.6% taper. The canals were rinsed with 3% NaOCl and saline and the smear layer was removed with 17% EDTA gel. All teeth were sterilized by autoclaving for 30 minutes at 121°C and the apical foramina were sealed with putty, with varnish applied to the root surfaces.

## Preparation of silver nanoparticles:

A reaction vessel was filled with 100 mL of a 0.01M of silver nitrate solution, followed by the addition of a solvent containing 0.1 g of Gallic acid and 10 mL of deionized water while being stirred magnetically. To raise the pH to11 of the reaction mixture, a 1.0M sodium hydroxidesolution was added immediately. After the pH was adjusted, the mixture was stirred for an additional 20 minutes [11].

## Preparation of probiotic irrigant:

Probiotics were extracted using microbiological processes (ATCC standards) in an aseptic environment. A conical flask received 15 mL of MRS broth and 1 g of probiotic capsule powder (BifilacGG mouth melt vanilla granules, Tablets India Ltd.) containing Lactobacillus rhamnosus. The mixture was vortexed for two minutes on Fisher Scientific TM Digital mixer, incubated at 37°C for 48 hours and then stored at 4°C. The produced probiotic samples were used within two weeks [12].

## Contamination with *E. faecalis*:

The 32 extracted teeth were grouped into 4 groups randomly with 8 teeth in each group.

[1] Group I - NaOCl irrigation

[2] Group II - probiotic solution irrigation

[3] Group III - silver nanoparticles solution irrigation

[4] Group IV - saline irrigation (negative control)

*E. faecalis* (*E. faecalis*; ATCC 19434) culture grown in BHI broth medium (53286 Sigma-Aldrich, USA) was used to infect the four groups. 1x 108 CFU/mL culture of Enterococus faecalis (*E. faecalis*), was inoculated in root canals of each previously sterilised sample after measuring by serial dilution and plating and were incubated for 21 days at 37°C and 95% humidity. Every 48 hours, the broth was replenished and gently aspirated out of the canal. After 21 days of incubation, all teeth in each group were irrigated with 5 mL of the respective irrigant using a 30-gauge double-vented needle. Post-irrigation samples were collected with 25.6% paper points and transported in vials containing 5 mL of nutrient broth. These vials were centrifuged for three intervals of 15 seconds each and subsequent dilutions (1:10, 1:100, 1:1000 and 1:10000) were made. A 50 µL volume from each dilution was plated in triplicate on Brain Heart Infusion agar. After 24 hours at 37°C and 95% humidity, *E. faecalis* colonies were counted and plates with the highest counts from each group's samples were selected.

## Results:

The data were analysed using IBM SPSS version 25. The intergroup comparison of post irrigation values among the four groups was done using ANOVA. The colony forming unit method revealed that there was statistically significant difference in the mean value of the *E. feacalis* count of 4 groups with the p value ≤.05 were considered significant (Table 1). The mean values of the *E. faecalis* count of probiotic irrigant (mean = 1.12) is lesser than that of silver nanoparticle irrigant (mean = 2.39)m. Intra group comparison was done by using post-hoc analysis showed that statistical significant difference was there between probiotic and the silver np group (p< 0.001).

## Discussion:

An effective endodontic treatment regimen begins with thorough root canal disinfection. *E. faecalis* biofilm is resistant to eradication as it has high ph toleration and can grow well in stress starvation and also higher salt content [13]. Numerous methods for biofilm removal include probiotics, functionalized nanoparticles (chitosan and silver), enzymatic agents and proteolytic agents like sodium hypochlorite and chlorhexidine [14]. In this study an attempt was made to compare the antimicrobial efficacy of probiotic and silver nanoparticle when used as irrigant against *E. faecalis* infection in extracted teeth. *E. faecalis* can colonize the root canal and infiltrate dentinal tubules, invading them after 21 days of incubation to form a structured biofilm. This timeframe mimics clinical scenarios, assuming proper decontamination. Colony-forming units (CFUs) were used for precise bacterial quantification in this study [15]. Results in the study have revealed that there is a significant difference between the antimicrobial efficacy of sodium hypochlorite, probiotic irrigant, silver nanoparticle irrigant and saline. Sodium hypochlorite showed the maximum antimicrobial efficacy (Table1) (mean = 0.58) followed by probiotic irrigant (mean value= 1.12), followed by silver nanoparticle irrigant (mean value = 2.39) and saline (mean value = 6.63). Sodium hypochlorite is the gold standard irrigant in endodontic treatment. This study used 5.25% sodium hypochlorite as a control. Its hyper tonicity removes fluid from cells due to osmotic pressure, while its ability to oxidize and hydrolyze proteins contributes to its antibacterial properties [16]. Sodium hypochlorite is cytotoxic to periapical tissue; therefore, non-cytotoxic and biocompatible alternatives like probiotic irrigants can be used in root canal treatments. The least difference in the antimicrobial efficacy was observed between the probiotic irrigant and the sodium hypochlorite groups whereas the maximum difference was observed between the saline irrigant and sodium hypochlorite irrigant. In the present study, the probiotic irrigant showed significant antimicrobial activity against *E. faecalis* which suggests that probiotics promote favourable anti-inflammatory effects thereby stopping the development of pathogenic activities of endodontic pathogens within a biofilm matrix [10]. Probiotics work by reducing inflammation, inhibiting collagenases and eliminating pathogens through the production of bacteriocins and other antagonistic compounds [18]. In another study it has been concluded that the probiotic irrigant can also be used as a safer alternative to sodium hypochlorite irrigant [17]. Numerous investigations have demonstrated that silver nanoparticles when used in the treatment of microorganisms which are resistant to antimicrobial drugs prevent the formation of biofilms because they attach and enter the cells of microorganism and discharges the silver ions which interfere with the cellular functioning [8]. However, in another study it was concluded that silver nanoparticle irrigant was ineffective against *E. faecalis*in root canal treatment [8]. Additionally, silver nanoparticles were tested as irrigants and medications. The irrigant did not significantly alter the biofilm or eradicate the bacteria, but the silver nanoparticle gel showed damage to the biofilms with lesser number of microbial count detected using CLSM [19, 20]. In this study the antimicrobial efficacy of silver nanoparticle is significantly lower than both sodium hypochloride and probiotic irrigant (p<0.001 in post hoc analysis).

## Conclusion:

Disinfection ensures positive outcome of root canal treatment resulting in long term success of treatment done and efficiency of clinician. Thus, the probiotic irrigant has better antimicrobial efficacy than silver nanoparticle irrigant against *E. faecalis*.

## Figures and Tables

**Table 1:** Post-hoc comparison of post irrigation values among the four groups

	**N**	**Mean**	**P value**	**Standard deviation**		**Mean difference**	**P value**
NaOCl	8	0.58	<.001	0.197	Probiotic	-0.537	0.002
					Silver NP	-1.812	<.001
					Saline	-6.051	<.001
Probiotic	8	1.12		0.171	Silver NP	-1.275	<.001
					Saline	-5.513	<.001
Silver NP	8	2.39		0.391	Saline	-4.238	<.001
Saline	8	6.63		0.223			
